# Physical symptoms among professional gamers within eSports, a survey study

**DOI:** 10.1186/s13102-024-00810-y

**Published:** 2024-01-15

**Authors:** Staffan Ekefjärd, Ramana Piussi, Eric Hamrin Senorski

**Affiliations:** 1 Department of Community Medicine and Rehabilitation, Physiotherapy, Umeå University, Umeå, Sweden; 2 Sahlgrenska Sports Medicine Center, Sahlgrenska Academy, Gothenburg, Sweden; 3 Department of Health and Rehabilitation, Unit of Physiotherapy, Institute of Neuroscience and Physiology, Sahlgrenska Academy, University of Gothenburg, Box 455, 405 30 Gothenburg, Sweden

**Keywords:** Symptoms, Gaming, Computer, Electronic sports

## Abstract

**Background:**

There is a need to establish the prevalence of self-reported physical symptoms such as pain in professional gamers (PGs) and to analyse whether there are correlations between lifestyle factors and self-reported physical symptoms. The purpose of this study was to analyse the prevalence of self-reported physical symptoms including eye-related problems in PGs. A further aim was to analyse the association between physical symptoms and lifestyle factors such as sleep time, play time and physical activity.

**Methods:**

This study was designed as a cross-sectional study with data based on an electronic survey, created specifically for this study, through discussion and screening of established validated questionnaires for physical symptoms in musculoskeletal conditions: the Karolinska Sleep Questionnaire and the Nordic Musculoskeletal Questionnaire. The survey comprised age and years of experience as a PG as descriptive variables, as well as questions on sleep, play time, physical activity and physical symptoms for the purpose of analysis. The directors of 10 professional gaming corporations were contacted by email with a link to the study-specific survey to distribute to all employees.

**Results:**

A total of 40 answers to the electronic survey were retrieved from 40 PGs, of which 62.5% (*n* = 25) had experienced at least one physical symptom in the three months prior to answering the survey. There was a significant association between playing time and physical symptoms (OR = 8.0; 95% CI 1.4–44.6, *p* = 0.018), where playing for more than 35 h a week was positively associated with eight times higher odds of experiencing physical symptoms.

**Conclusion:**

There is a high prevalence of physical symptoms, such as headache and eye symptoms, in professional eSports gamers. There was an association between playing more than 35 h per week with the prevalence of physical symptoms.

**Supplementary Information:**

The online version contains supplementary material available at 10.1186/s13102-024-00810-y.

## Introduction

Electronic sports (eSports) is the definition used for competitions in video games (computer or console). Individuals who are registered in professional gaming corporations and compete in eSports are called professional gamers (PGs). ESports grew exponentially during the 2010s, and 24 countries have recognised eSports as an official sport [1].

Being a PG means that several hours each day are spent on sedentary work in front of a computer. With regard to physical activity within eSports, up to 90% of PGs reported only being physically active for one hour a day on average [2]. A survey study of German PGs showed that 50% of the gamers trained for five hours a week [3]. These reported low levels of physical activity lead to the question of whether PGs develop musculoskeletal conditions, such as pain. With the rapid increase in popularity of eSports, the relationship between eSports and health-related outcomes has attracted more attention. Since 2018, gaming disorder (GD) has been included in the World Health Organisation (WHO) diagnosis classification (ICD-11) [4, 5]. Accordingly, GD is characterised by reduced control of the gaming activity, where gamers prioritise gaming before other interests or daily activity and continue to game despite negative consequences such as physical symptoms, including pain or neurological symptoms, i.e. symptoms related to sight and the ability to concentrate [4, 5]. Analyses of health and lifestyle factors in PGs in the US (*n* = 65) reported that 56% of gamers had eye tiredness, 42% had pain in the neck and back, 36% had pain in the wrist and 32% had pain in the hand [6]. Since GDs involve the prioritisation of gaming before other activities, sleep is one of the activities at risk of being compromised. Sleep is a key lifestyle factor for function and well-being and sleep disturbance might lead to new or increased pain conditions [7], as short sleep time might infer higher tissue sensitivity towards pain [8].

Systematic reviews of sedentary computer workers have reported that equipment (keyboard) placement, insufficient pauses (under three to five minutes every working hour) and repetitive work in front of a computer can increase the risk of developing musculoskeletal disorders [9–11], as well as eye-related problems, such as excessive dryness [12, 13].

Some ways to prevent upper extremity musculoskeletal disorders are resistance training, exercise interventions and exercise therapy, which have been reported with moderate to strong evidence as a preventive interventions for different upper extremity musculoskeletal disorders [9, 14–16]. On the other hand, arm support with alternative mouse was the only ergonomic intervention that reduced pain [17], while other ergonomic interventions [17] or workplace interventions benefits for pain relief in computer workers were neither supported nor refused [18]. The incidence of neck or shoulder and right upper limb disorders were not considerably reduced by ergonomic interventions [19]. Since the field of professional gaming is rapidly growing, there is a need to study health related problems in PGs. Further, there is a need to explore the prevalence of physical symptoms such as pain in PGs and to analyse whether there are associations between lifestyle factors, such as sleep time, play time, physical activity, and physical symptoms.

The purpose of this study was to analyse the prevalence of self-reported physical symptoms, including eye-related problems, in professional gamers. A further aim was to analyse the association between physical symptoms and lifestyle factors, such as sleep time, play time and physical activity.

## Method

This study was performed according to the Strengthening The Reporting of Observational Studies in Epidemiology (STROBE) statement [20]. This study was conducted in Sweden and designed as a cross-sectional study with data based on an electronic survey sent out in January 2020. A survey specific to this study (Additional file 1: Table 1) was created through discussion and screening of established validated questionnaires for physical symptoms in musculoskeletal conditions [21–23].

### Survey construction

When creating the survey, responder burden was taken into account and the survey was not supposed to take longer than 10 min to answer. The survey was created in Swedish and translated to English by two professional copywriters, with the exception of the questions taken from the Nordic Musculoskeletal Questionnaire (NMQ), which were taken from the English version [23]. First, one copywriter translated from Swedish to English and then the second translated the English version back to Swedish in order to ensure nothing was erroneusly missed or wrongly translated. The final English version was administered to 10 amateur gamers in order to assess comprehensibility. No revisions to the survey were made. A free online survey service: “Surveymonkey” (https://sv.surveymonkey.com/) was used for the administration of the survey. Surveymonkey does not keep trace of the IP adress which ensures anonymity.

The survey comprised age and years of experience as a PG as descriptive variables and questions about sleep, playing time, physical activity and physical symptoms for the purpose of analysis. With regard to sleep, two questions about time of going to bed and time of waking up were taken from a valid and reliable questionnaire: the Karolinska Sleep Questionnaire (KSQ) [21]. We used two questions on physical actitity with good reliability and validity, which are recommended by the Swedish National Board of Health and Welfare [22]. PGs responded to multiple-choice questions on the time spent on a certain activity in a regular week. The first question on physical activity asked PGs how much time they spend on physical exercise that causes them to be out of breath. The second question on physical activity asked PGs how long they spend on daily exercise such as walking, cycling, or gardening. The difference between physical exercise and daily exercise was the exertion level, where physical exercises refer to physical activity that caused PGs to be out of breath. Self-reported weekly playing time was recorded by a multiple-choice question with eight different option (fewer than 15 h; 15–20 h; 20–25 h; 25–30 h; 30–35 h; 35–40 h; 40–45 h; over 45 h). Questions relating to the presence of musculoskeletal symptoms were taken from the NMQ [23], which has good to moderate reliability between questionnaire answers and interviews (kappa values between 0.50 and 0.68) [24]. Since eye symptoms and headache were found to be common among PGs [6] we chose to add head (headache) and eyes (symptoms from the eyes, for example tired, dry, scratchy) as possible locations for physical symptoms in the study-specific survey. Thus, in the present study, physical symptoms were defined as pain reported in any of the following body regions: fingers; hands; wrists; elbows; shoulders; neck; lower back; head (headache); eyes (symptoms from the eyes, for example tired, dry, scratchy). The PGs were asked to answer dichotomously (yes/no) whether they had any symptoms, i.e. pain, in the last three months, as opposed to 12 months in the NMQ, for each body part. Finally, PGs were then asked whether they had sought medical care for the symptoms, in order to judge the severity of symptoms [6].

### Participants

PGs able to write and read English, under contract to professional gaming corporations, were eligible for inclusion. Since the PGs’ gaming corporations can employ PGs online, inclusion criteria were not limited to geographical areas.

The directors of 10 professional gaming corporations (randomly chosen from an analytical agency collecting all information about eSports and streaming: http://escharts.com) were contacted by email in January 2020 and informed about the study. No strict definition of professional gaming corporation was used. The directors of gaming corporations were provided with a link to the study-specific survey to distribute to all employees, i.e. PGs. The PGs received written information about the study, the purpose, the fact that participation was voluntary and that all collected data would be treated anonymously and reported at group level. No incentives for participation were announced. The study was performed in accordance with the Declaration of Helsinki. Written informed consent was obtained. The Swedish Ethical Review Authority was contacted and deemed that the project did not require ethical approval since no intervention or collection of personal data would take place.

### Data analysis

Statistical analysis was performed with the Statistical Package for Social Sciences (SPSS) (version 26, SPSS Inc., Chicago, IL, USA). A significance level of 0.05 was set. A logistic regression model was used to determine the association between outcome of interest (presence of physical symptoms) and variables assessed (sleep, physical activity, time spent playing). Due to the small number of responses (*n* = 40), descriptive variables were dichotomised: for physical activity, PGs were grouped into “reaching 150 min/week (yes/no)” [22]; to calculate whether a PG reached 150 min per week, the sum of minutes reported both to the question “how much time PGs spend on physical exercise that causes them to be out of breath” and to the question “how long PGs spend on daily exercise such as walking, cycling, or gardening “ was calculated. For sleep, PGs were grouped into “sleep fewer than eight hours (yes/no), as reported in the study by Brauer et al. [8] as a public health recommendation. For playing time, PGs were dichotomised into “plays more than 35 h/week” (yes/no) to reflect a full-time working week (35 h were chosen to take account of the fact that not all 40 working hours are in front of the screen. For example, meetings might take time from the playing schedule).

## Results

A total of 40 answers to the electronic survey were retrieved from 40 PGs. Demographic and descriptive variables are presented in Table 1).

**Table 1:** Demographic and descriptive variables

**Age (years); % (n)**	
Under 18	0% (*n* = 0)
18–22	47.5% (*n* = 19)
23–27	32.5% (*n* = 13)
28 or older	20% (*n* = 8)
**At what time do you usually go to bed? (median, min;max)**	11p.m. (10p.m; 3.30a.m.)
**At what time do you usually wake up (median, min;max)**	7a.m. (5.30a.m; 12.30a.m.)
**Hours of sleep; mean; range (median, min;max)**	8.20; 5–13 (8, 5;13)
**Years as a professional gamer**	
5 years or less	80% (*n* = 32)
More than 5 years	20% (*n* = 8)
**Time spent on physical exercise that causes you to be out of breath % (n)**	
None	7.5% (*n* = 3)
< 30 min	22.5% (*n* = 9)
30–60 min	22.5% (*n* = 9)
60–90 min	10% (*n* = 4)
90–120 min	10% (*n* = 4)
> 120 min	27.5% (*n* = 11)
**Time spent on daily exercise % (n)**	
None	0% (*n* = 0)
< 30 min	7.5% (*n* = 3)
30–60 min	30% (*n* = 12)
60–90 min	25% (*n* = 10)
90–150 min	7.5% (*n* = 3)
150–300 min	10% (*n* = 4)
> 300 min	20% (*n* = 8)
**Time spent on screen in an average week? % (n)**	
> 15 h	0% (*n* = 0)
15–20 h	2.5% (*n* = 1)
20–25 h	2.5% (*n* = 1)
25–30 h	7.5% (*n* = 3)
30–35 h	10% (*n* = 4)
35–40 h	22.5% (*n* = 9)
40–45 h	30% (*n* = 12)
> 45 h	25% (*n* = 10)

*min* minutes, *h* hours, *n* number

**Fig. 1:**
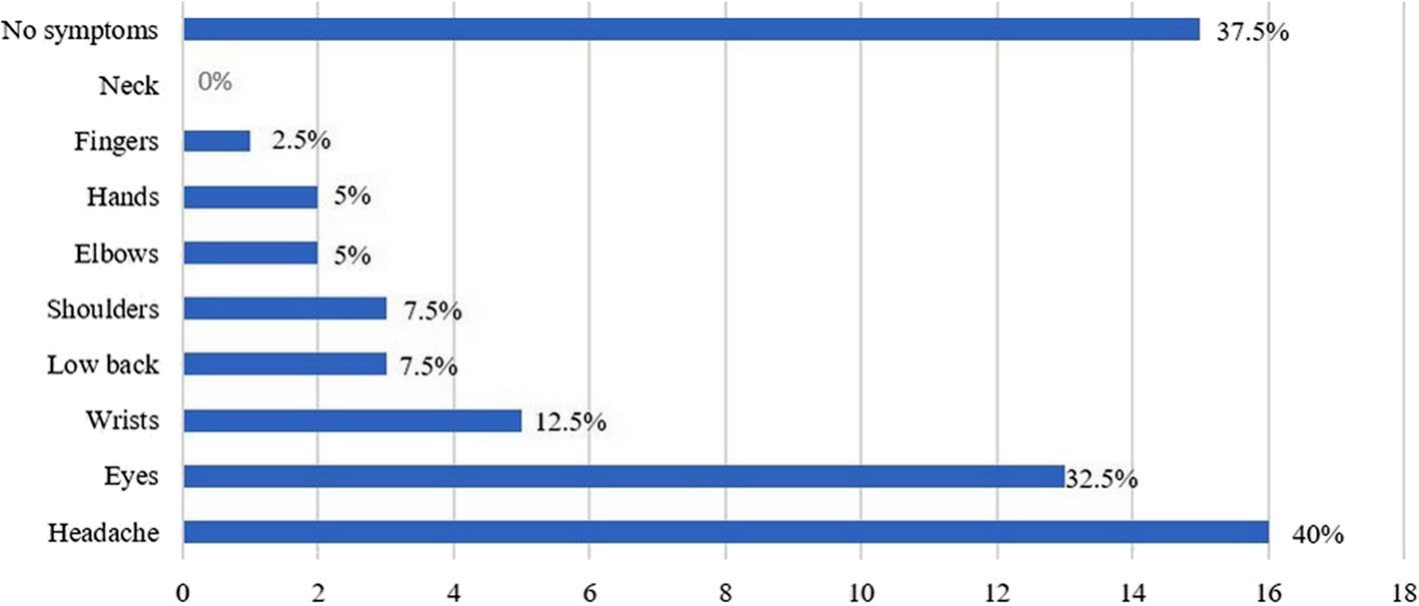
Point prevalence of physical symptoms in professional gamers

Among the PGs, 37.5% (*n* = 15) had not experienced any physical symptom associated with gaming in the three months before taking the survey, while 62.5% (*n* = 25) experienced at least one physical symptom (Fig. 1). Of the 25 PGs who reported physical symptoms, the vast majority (22/25, 88%) experienced physical symptoms in one or two body areas (Table 2). Two PGs (5%) had sought healthcare because of the physical symptoms they experienced.

**Table 2:** Odds ratio between sleep, physical activity and time spent playing with musculoskeletal symptoms, including eye-related problems

	OR	95% CI	*P* value
Sleeping more than eight hours	0.722	0.132–3.965	0.708
Playing more than 35 h a week	8.012	1.439–44.261	0.018†
More than 150 min physical activity	1.004	0.225–4.482	0.996

*OR* Odds Ratio, *CI* Confidence Interval

†*p* < 0.05

**Fig. 2:**
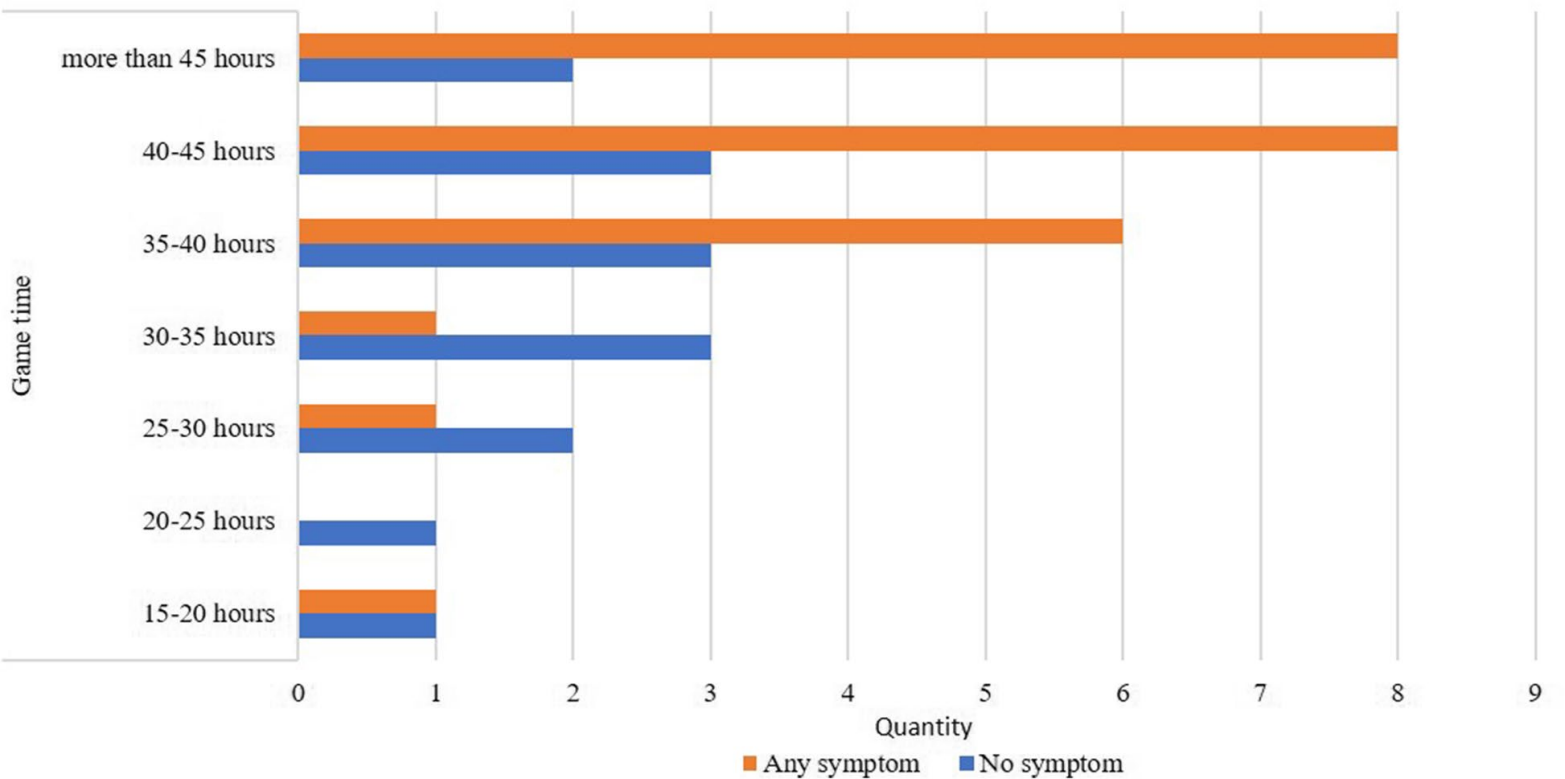
Experienced physical symptoms stratified for playing time

Among the PGs, 47.5% (*n* = 19) met the Swedish National Board of Health and Welfare’s recommendations for physical activity [22] of at least 150 min per week. Furthermore, 67.5% (*n* = 27) of the participants reported sleeping at least eight hours a night (median 8, min–max 5–13). Thirty of the PGs (75%) reported playing for at least 35 h per week and, among those who play for more than 45 h a week (*n* = 10), 80% reported experiencing physical symptoms (Fig. 2).

Table 2 presents the results from the binary logistic regression model of the presence of physical symptoms and sleep, physical activity and time spent playing. There was a significant association between playing time and physical symptoms (OR = 8.0; 95% CI 1.4–44.6, *p* = 0.018), where playing for more than 35 h a week was positively associated with eight times higher odds of experiencing physical symptoms.

## Discussion

The main finding in this cross-sectional survey study was that the prevalence of self-reported physical symptoms in professional eSports gamers was 62.5%. There was a positive association between playing more than 35 h per week with the prevalence of physical symptoms. On the other hand, there was no association between physical symptoms and sleep time or physical activity.

Playing for more than 35 h a weeks resulted in eight times higher odds of perceiving physical symptoms. The association between a long playing time and a higher prevalence of physical symptoms has been confirmed and previously reported in the literature [11, 25, 26]. An explanation for the association between pain and extended screen time could be the absence of significant body movement, potentially leading to pain. Additionally, evidence suggests that both individual and psychosocial factors contribute to musculoskeletal pain development, where fewer social contacts have a favourable effect on the development of pain [27, 28]. In theory, prolonged computer use among PGs might affect their engagement in social interactions, potentially leading to increased physical symptoms like pain. In light of the association between screen time and development of physical symptoms, stakeholders interested in the well-being of PGs should consider implementation of strategies that encourage breaks during extended gaming sessions.

In sedentary workers, prevalence of physical symptoms such as pain ranged between 43.1% and 93% in professional drivers [29]. Pain is more common with prolonged use of keyboards, and in computer workers, prevalence of pain varies greatly, with office workers showing trends of higher prevalence compared with nurses and other workers [30]. Looking at the general Swedish population, the prevalence of pain appears to increase with age (odds of having pain became about one and a half times higher for each decade people age) and differs from decade to decade, with the a prevalence range from 39% (in 1968) to 62% (in 2000) [31].

The comparison of the prevalence of pain with that of the general population proves challenging due to variations in pain epidemiology across geographical areas and shifts in prevalence over time. These factors make direct comparisons notably difficult [32]. Furthermore, changes to the questions asked or definitions used, albeit small, can have an important effect on the prevalence [32]. Comparing prevalence between countries is even more problematic, as observed differences could be influenced by language [32].

Headache was reported as being the most common physical symptoms, prevalent in 40% of the PGs in this study. Headache is reported as a common symptom among computer workers [12, 13]. In comparison, 22% of the Swedish population between 16–29 years of age reported to have headaches in 2018 [33]. In the general population, the overall prevalence of primary headaches is reported as 58.4% [34]. Through the Global Burden of Diseases, Injuries and Risk Factors (GBD) studies, headaches have emerged as a major global public health concern, as important causes of disability worldwide [35]. The therapeutic exercise management of headaches has proven beneficial in reducing headache frequency and intensity [36, 37]. Prolonged periods spent in front of computer screens may contribute to the prevalence of headaches. Therefore, stakeholders, such as corporations employing PGs, could benefit from collaborating with healthcare providers experienced in the administration of exercise therapy to minimise the burden of headache in PGs.

The prevalence of physical symptoms in this study is within the same interval as that reported in other sedentary professions such as musicians (39–68%), inferring that computer work does not induce more pain than other sedentary work [38, 39]. Prolonged standing in front of a screen may induce Computer Vision Syndrome, which encompass symptoms like eyestrain, blurred vision, dry eyes, and headaches [40]. According to a previous publication on eSports [6], eye-related problems such as dryness or blurred vision had a high prevalence (56%) and, in our study, the prevalence of eye-related problem was 32.5%. However, 32.5% is lower than the prevalence of eye-related problems reported among computer workers, 64–90% [12, 13]. Effective prevention of Computer Vision Syndrome involves measures such as light adjustments, ergonomic considerations, and patient education. Education includes advising individuals to take frequent short breaks while working in front of a screen, which has shown significant effectiveness in reducing Computer Vision Syndrome prevalence [40]. Short pauses between each match or game session, or to be genetically predisposed to long playing sessions could be some of the reasons for a lower prevalence of eye-related problems in PGs compared with computer workers. Notably, no PGs reported to suffer from neck pain using questions from the NMQ. Neck, shoulders and upper back were the body parts with highest prevalence of pain in air controllers [41]. Similarly, computer workers in Nigeria and Australia reported the highest prevalence of pain in neck and back [42, 43]. It is unknown why no professional PGs reported neck pain. One potential explanation could be attributed to the survey's lack of visual aids, unlike the NMQ, where respondents can precisely identify the affected area. Larger studies are required to validate or refute this observation.

In our results, almost half (47.5%) of PGs reached the recommended 150 min of physical activity a week. The percentage is relatively low when considered in comparison to the general Swedish population between 16–29 years of age, where, in 2018, 72% reached the recommended 150 min a week [33]. Other studies on PGs revealed that 60% reached the recommendation and that PGs exercised for more than 1.08 h a day on average [2, 3]. Reaching 150 min of physical activity per week or not did not correlate with the prevalence of physical symptoms. Consequently, in our cohort, the level of physical activity did not seem to influence the development of physical symptoms.

The median sleeping time for PGs in Germany has been reported to be 7.9 h [3], which is in line with that of our cohort (median hours of sleep = 8). Furthermore, 28.3% of adults sleep for six or fewer hours, 8.5% sleep for nine or more hours and 63.3% sleep for seven or eight hours according to a population-based study from the US [44]. Consequently, the PGs in our study appear to sleep as much as the majority of adults in the US. Nevertheless, the majority of included PGs were 18–27 years of age, which is substantially younger than the mean age (45 years) of US adults in Krueger et al. [44] Negative correlations between hours of sleep and the prevalence of pain have previously been reported [7, 45, 46]. In our study, we therefore expected a similar relationship. However, we did not find any association between sleeping duration and the prevalence of physical symptoms. The absence of association might be due to the limited number of participants in our study or the wide range of sleeping hours [5–13]. Moreover, solely measuring sleeping hours does not provide insights into sleep quality, including whether participants feel fully rested or not after a sleep session.

## Limitations

The most significant limitation in our study is the small number of responses to the survey. The limited number of PGs led to grouping certain responses into dichotomous variables, affecting the statistical power of our study and restricting the generalizability of our findings. While almost 40% of PGs in this study did not report pain, it is plausible that more PGs experiencing pain opted to join the study, suggesting potential participation bias. Readers are advised to interpret our results cautiously. Another limitation is the inability to stratify results by sex, as studies indicate that women tend to experience pain more frequently than men [47]. As eSports is a sex-neutral sport where all PGs compete on equal terms, we chose not to include sex in the survey. However, males are in general over represented within eSports [2, 48] and this therefore limits the generalisability of our results. Another limitation in our findings is the lack of consideration for symptom severity. Given that only 5% of the surveyed PGs sought medical care, it is plausible that our questionnaire lacked the sensitivity to stratify physical symptoms based on their severity levels. Consequently, our study results should be approached cautiously, as they may not fully capture the nuanced complexity of pain experienced among PGs. Nevertheless, considering the rapid increase in the popularity of eSports, digitalisation in society and the association between playing time and the prevalence of physical symptoms, PGs require tailored prevention programmes, interventions and further knowledge of the risk factors for pain. Lastly, the survey was not tested for validity or reliability, and PGs were asked to report where they perceived pain, but no body figure was provided to verify the area of pain, as it is provided in the NMQ.

## Conclusion

There is a high prevalence of physical symptoms, such as headache and eye symptoms, in professional eSports gamers. There was an association between playing more than 35 h per week with the prevalence of physical symptoms.

### Supplementary Information

**Additional file 1: Table 1.** Survey

## Data Availability

The datasets used and/or analysed during the current study are available from the corresponding author on reasonable request.

## Abbreviations

PGs: Professional gamers
eSports: Electronic sports
GD: Gaming disorder
WHO: World health organisation
STROBE: Strengthening the reporting of observational studies in epidemiology
KSQ: Karolinska sleep questionnaire
NMQ: Nordic musculoskeletal questionnaire
SPSS: Statistical package for the social sciences
