# Supplementary material for: Physical symptoms among professional gamers within eSports, a survey study
Source: BMC Sports Sci Med Rehabil. 2024 Jan 15;16:18. doi: 10.1186/s13102-024-00810-y (PMC10790447; doi:10.1186/s13102-024-00810-y)
Supplement: Supplementary file 1 — Additional file 1: Table 1. Survey [file 13102_2024_810_MOESM1_ESM.docx]

**Additional file**

| Addiitonal file 1: Table 1. Survey | |
| --- | --- |
| In the following survey, you will answer questions about health and lifestyle. The aim is to examine the presence of physical symptoms among professional esports athletes and to determine whether there is a correlation between physical activity, screen time, sleep and physical symptoms. Participating in this study is voluntary and anonymous. By responding to the survey, you agree to participate in this study.  Select the option that best matches your situation | |
| Age | A. Under 18 |
|  | B. 18-22 |
|  | C. 23-27 |
|  | D. 28 or older |
| Number of years as a professional esports athlete? | A. Five years or less |
|  | B. More than five years |
| In a regular week, how much time do you usually spend on physical exercise that causes you to be out of breath? (for example, running, aerobics or ball sports) | A. 0 minutes/no time |
|  | B. Fewer than 30 minutes |
|  | C. 30-60 minutes (0.5-1 hour) |
|  | D. 60-90 minutes (1-1.5 hours) |
|  | E. 90-120 minutes (1.5-2 hours) |
|  | F. More than 120 minutes (2 hours) |
| In a regular week, how much time do you usually spend on daily exercise such as walking, cycling, or gardening? Add up the time (at least 10 minutes at a time) | A. 0 minutes/no time |
|  | B. Fewer than 30 minutes |
|  | C. 30-60 minutes (0.5-1 hour) |
|  | D. 60-90 minutes (1-1.5 hours) |
|  | E. 90-150 minutes (1.5-2.5 hours) |
|  | F. 150-300 minutes (2.5-5 hours) |
|  | G. More than 300 minutes (5 hours) |
| At what time do you usually go to bed (turn off the lights)? |  |
| At what time do you usually wake up? |  |
| In a regular week, how much time do you usually spend on computer and/or video games? | A. Fewer than 15 hours |
|  | B. 15-20 hours |
|  | C. 20-25 hours |
|  | D. 25-30 hours |
|  | E. 30-35 hours |
|  | F. 35-40 hours |
|  | G. 40-45 hours |
|  | H. More than 45 hours |
| In the last three months, have you experienced physical symptoms in any of the following body parts? (This can be symptoms such as pain, aching, discomfort, numbness etc.) | A. Fingers yes/no |
|  | B. Hands yes/no |
|  | C. Wrists yes/no |
|  | D. Elbows yes/no |
|  | E. Shoulders yes/no |
|  | F. Neck yes/no |
|  | G. Lower back yes/no |
|  | H. Head (headache) yes/no |
|  | I. Eyes (symptoms from the eyes, for example tired, dry, scratchy) yes/no |
|  | J. Other yes/no |
| Have you sought medical care for your symptoms? | A. Yes |
|  | B. No |
